# Excess mortality among people in homelessness with substance use disorders: a Swedish cohort study

**DOI:** 10.1136/jech-2023-220989

**Published:** 2024-05-21

**Authors:** Sophie Nadia Gaber, Johan Franck, Härje Widing, Jonas Hällgren, Elisabet Mattsson, Jeanette Westman

**Affiliations:** 1 Department of Healthcare Sciences, Marie Cederschiöld högskola—Campus Ersta, Stockholm, Sweden; 2 Department of Women's and Children's Health, Healthcare Sciences and e-Health, Uppsala University, Uppsala, Sweden; 3 Faculty of Brain Sciences, Division of Psychiatry, University College London, London, UK; 4 Department of Clinical Neuroscience, Karolinska Institutet, Stockholm, Sweden; 5 Academic Primary Care Center, Region Stockholm, Stockholm, Sweden; 6 Department of Neurobiology, Care Sciences and Society, Karolinska Institutet, Stockholm, Sweden

**Keywords:** SUBSTANCE ABUSE, EPIDEMIOLOGY, HOMELESS PERSONS, MORTALITY

## Abstract

**Background:**

People in homelessness have an increased risk of substance use disorders (SUDs) and poor health outcomes. This cohort study aimed to investigate the association between homelessness and mortality in people with SUDs, adjusting for age, sex, narcotic use, intravenous drug use and inpatient care for SUDs.

**Methods:**

Data from the Swedish National Addiction Care Quality Register in the Stockholm region were used to analyse mortality risk in people with SUDs (n=8397), including 637 in homelessness, 1135 in precarious housing and 6625 in stable housing, at baseline. HRs and CIs were calculated using Cox regression.

**Results:**

Mortality was increased for people in homelessness (HR 2.30; 95% CI 1.70 to 3.12) and precarious housing (HR 1.23; 95% CI 0.86 to 1.75) compared with those in stable housing. The association between homelessness and mortality decreased (HR 1.27; 95% CI 0.91 to 1.78) after adjusting for narcotic use (HR 1.28; 95% CI 1.00 to 1.63), intravenous drug use (HR 1.98; 95% CI 1.52 to 2.58) and inpatient care for SUDs (HR 1.96; 95% CI 1.57 to 2.45). Standardised mortality ratios (SMRs) showed that mortality among people in homelessness with SUDs was 13.6 times higher than the general population (SMR=13.6; 95% CI 10.2 to 17.9), and 3.7 times higher in people in stable housing with SUDs (SMR=3.7; 95% CI 3.2 to 4.1).

**Conclusion:**

Homelessness increased mortality, but the risk decreased after adjusting for narcotic use, intravenous drug use and inpatient care for SUDs. Interventions are needed to reduce excess mortality among people in homelessness with SUDs.

**What is already known on this topic**

- People in homelessness have an increased risk of substance use disorders (SUDs). SUDs are associated with an increased risk of mortality.
- People in homelessness have an increased risk of excess mortality but the association between excess mortality and SUDs requires further investigation.

**What this study adds**

- Few previous studies have investigated the association between homelessness and SUDs in a large cohort study.
- Among the study population, the risk of mortality among people in homelessness at baseline was higher than among people in stable housing.
- The increased mortality risk among people in homelessness with SUDs was no longer significant after adjusting for narcotic use, intravenous drug use and inpatient care for SUDs.

**How this study might affect research, practice or policy**

- This study underscores the need for policies and targeted interventions to support people in homelessness with SUDs to decrease excess mortality in this vulnerable population.

## Introduction

People in homelessness have an increased risk of mortality.^1–3^ An estimated 700 000 people are homeless in Europe,^4^ of which approximately 30 000 are in Sweden.^5^ The intersection of physical and mental health issues, and substance use disorders (SUDs), disproportionately impacts people in homelessness.^1 6^ Homelessness has consistently been associated with poor health outcomes^1 6 7^ and excess mortality.^2 8–15^ In comparison to the general population, previous studies have calculated standardised mortality ratios (SMRs) between 2.0 and 6.7 for people in homelessness.^1 2 9–17^ However, more knowledge is needed to understand if the problem is homelessness itself and to what extent other factors contribute to excess mortality.^2 8 14^

Elevated risks of mortality among people in homelessness have been partly explained by exposure to risk factors, such as smoking, alcohol, substance use^1 8 10 18^ and psychiatric disorders,^8 12 15 19^ which may coexist.^1 2 20^ Psychiatric disorders are more prevalent among people in homelessness compared with the general population, with SUDs being the most common psychiatric disorder among this population.^1 8 19^ Inpatient admissions for psychiatric disorders and SUDs are higher among people in homelessness compared with those not in homelessness.^14^ Furthermore, the year following discharge from inpatient care is a high-risk period for homelessness.^21^

Intravenous drug use has been associated with an increased risk of mortality among people in homelessness.^3 22–24^ Homelessness potentiates relapse to drug use and intravenous drug-related risk behaviour,^22^ in addition to the risk of HIV^23 24^ and hepatitis C virus transmission in people using intravenous drugs.^23^

Systematic reviews on mortality among people in homelessness^3 25^ reveal that the majority of studies have been conducted in North America,^3 11–13 18 26–29^ or are based on older data collections^15 16 19^; thus, there is a need to develop up-to-date insights from other regions. This study uses data from the Swedish National Addiction Care Quality Register (Swedish name: Bättre Beroendevård) which records housing status and provides a new and unique opportunity to perform research among people in homelessness or precarious housing who have SUDs (hereafter, quality register).

## Aim

This cohort study aimed to investigate the association between homelessness and mortality in people with SUDs, adjusting for age, sex, narcotic use, intravenous drug use and inpatient care for SUDs.

## Methods

Data were retrieved from the quality register. The purpose of the quality register is to contribute to improved quality and more equal addiction care nationwide by measuring quality indicators.^30^ Inclusion criteria for the quality register are that individuals must have a SUD diagnosis, as defined by the International Classification of Diseases, 10th Revision (ICD-10 codes, F10–F19^31^), and be receiving treatment at a specialised addiction care clinic.

For this study, we used data from the Stockholm healthcare region where the quality register is integrated into electronic healthcare record systems; thus, patient data are directly transferred to the quality register with a high coverage rate.^30^ The quality register data encompasses a range of information, including sociodemographic characteristics, diagnoses, pharmaceutical treatment, psychosocial treatment, self-reported substance use and outcomes. All data are collected by healthcare staff during healthcare visits to specialised addiction centres. Through structured interviews, the healthcare staff use predefined terms to ask the patient about various questions, including the patient’s intravenous drug use and substance use.

The quality register is updated about whether a patient is alive or deceased from national statistics (Statistics Sweden) daily. The mortality data in the quality register are sourced from the Swedish National Death Register. Linkage between different data sources (ie, the quality register and the Swedish National Death Register) was performed on the individual level using the personal identification number (PIN). PINs, which are unique for all Swedish citizens and permanent residents, are used in all Swedish national health and sociodemographic registers.

### Study population

The cohort consisted of 8397 patients with SUDs that fulfilled the inclusion criteria. Table 1 presents the baseline characteristics of the people in homelessness (n=637), in precarious housing (n=1135) or in stable housing (n=6625). Inclusion criteria for the study were patients: (1) aged 18 years and over with a SUD diagnosis (ICD-10 codes, F10–F19)^31^ ; and (2) registered in the quality register with their housing status between 2018 and 2020. The inclusion date refers to the first recorded housing status. The start of follow-up was at the inclusion date (ie, 2018–2020) and follow-up continued until death, or 31 December 2022. Baseline information including psychiatric diagnoses and self-report variables was collected at baseline and 365 days prior (ie, information that is registered during these 365 days). Other than mortality data, no data pertaining to events beyond baseline are included in the analyses.

**Table 1:** Baseline characteristics of the study population (n=8397)

	Homelessness	Precarious housing	Stable housing	P value
	n=637	n=1135	n=6625	All groups
Sex, female	163 (25.6%)	291 (25.6%)	2361 (35.6%)	<0.001
Age at inclusion, mean (SD)	40.7 (12.6)	33.9 (12.1)	47.1 (14.0)	<0.001
Opioid use disorder (ICD-10 F11)	215 (33.8%)	173 (15.2%)	916 (13.8%)	<0.001
Alcohol use disorder (ICD-10 F10)	245 (38.5%)	480 (42.3%)	4557 (68.8%)	<0.001
Other SUDs (ICD-10 F12–F16, F18–F19)	447 (70.2%)	677 (59.6%)	1700 (25.7%)	<0.001
Psychiatric comorbidity	319 (50.1%)	495 (43.6%)	2459 (37.1%)	<0.001
Substance use	576 (90.4%)	985 (86.8%)	5378 (81.2%)	<0.001
Narcotic use	467 (73.3%)	681 (60.0%)	1913 (28.9%)	<0.001
Intravenous drug use	246 (38.6%)	165 (14.5%)	574 (8.7%)	<0.001
Alcohol use	356 (55.9%)	682 (60.1%)	4541 (68.5%)	<0.001
Inpatient care for SUDs	341 (53.5%)	262 (23.1%)	1071 (16.2%)	<0.001
Opioid maintenance treatment	149 (23.4%)	91 (8.0%)	524 (7.9%)	<0.001
Psychosis (ICD-10 F20–F29)	42 (6.6%)	42 (3.7%)	134 (2.0%)	<0.001
PTSD (ICD-10 F43.1)	27 (4.2%)	46 (4.1%)	163 (2.5%)	0.001

ICD-10, International Classification of Diseases, 10th Revision; PTSD, post-traumatic stress disorder; SUDs, substance use disorders.

### Variables

Table 2 presents an overview of the key variables included in the analyses.

**Table 2:** List of variables

Variable	Items	Variable information
Sex	Male=1 Female=2	Sex was defined as male or female.
Age	Continuous	Age was calculated at baseline.
Housing status	Homelessness=1 Precarious housing=2 Stable housing=3	Patients were asked the following question at the addiction clinic: “how was your living situation in the last 30 days?” Housing status was based on three predefined groups: homelessness, precarious housing and stable housing.
Substance use disorders (SUDs)	Yes=1 No=0	Yes: Mental and behavioural disorders due to use of: opioids (ICD-10 F11); alcohol (ICD-10 F10); mixed substances including cannabinoids, sedatives or hypnotics, cocaine, stimulants including caffeine, hallucinogens, volatile solvents, multiple drug use and use of other psychoactive substances (ICD-10 F12–F16, F18–F19).
Narcotic use	Yes=1 No=0	Yes: Used any narcotics in the last 30 days, including heroin, methadone, mono-buprenorphine, buprenorphine-naloxone, other opioids, cocaine, amphetamines, stimulants with hallucinogenic effects, other narcotic stimulants, benzodiazepines, zolpidem, zopiclone or zaleplon, other narcotic depressant substances, hallucinogens, cannabis, synthetic cannabinoids, solvent, GHB, anabolic androgenic steroids, other performance-enhancing substances.
Intravenous drug use	Ever=1 Never=0	Ever: (i) injected drugs previously but not during the last 12 months; (ii) injected drugs during the last 12 months but not during the last 30 days; or (iii) injected drugs during the last 30 days. Never: no, never injected drugs.
Inpatient care for SUDs	Yes=1 No=0	Yes: ≥1 day of specialised residential treatment for SUDs (ICD-10 F10–F19) in the 12 months prior to baseline.
Selected psychoactive drug use	Yes=1 No=0	Yes: Used psychoactive drugs in the 12 months prior to baseline including other narcotic stimulants, other opioids, buprenorphine-naloxone, hallucinogens, heroin, methadone, mono-buprenorphine, stimulants with hallucinogenic effects.
Psychiatric comorbidity	Yes=1 No=0	Yes: Substance use disorder (ICD-10 F10–F19) combined with another psychiatric diagnosis (ICD-10 F00–F09, F20–F99).
Psychosis	Yes=1 No=0	Yes: Psychosis including schizophrenia, schizotypal and delusional disorders (ICD-10 F20–29).
PTSD	Yes=1 No=0	Yes: Post-traumatic stress disorder (ICD-10 F43.1).
Mortality		Date of death.
Causes of death		
Alcohol related	Yes=1 No=0	Causes of death were based on information from the Swedish Death Register. Causes of death were coded according to the ICD-10. If both alcohol-related and drug-related causes of death were registered, then the death was classified as drug related. Yes: Alcohol-related cause of death (ICD-10 E244, F10, G312, G621, G721, I426, K292, K700-K704, K709, K852, K860, O354, P034, Q860, R780, T510, Y90-Y791, Z714, Z721). No: Both alcohol-related and drug-related cause of death, or non-alcohol-related cause of death.
Drug related	Yes=1 No=0	Yes: Drug-related cause of death (ICD-10 F11–F16, F18–F19, O355, P044, T40, T436, Z503, Z715, Z722).
External causes*	Yes=1 No=0	Yes: External causes of death (ICD-10 V00–V99, X00–X59, X60–X84, Y10–Y34).
Other causes	Yes=1 No=0	Yes: Other ICD-10 codes than those included for alcohol related, drug related or external causes of death.
Unknown causes	Yes=1 No=0	Yes: ICD-10 code unknown at the time of data collection.

*External causes include suicide, unknown suicide, accident or assault. ICD-10 coding according to the WHO’s International Statistical Classification of Diseases and Related Health Problems (10th edition).^31^

ICD-10, International Classification of Diseases, 10th Revision; PTSD, post-traumatic stress disorder.

### Statistical analysis

Statistical comparisons between groups were made using χ^2^ tests for categorical variables and the analysis of variance for the continuous variable, that is, age (see tables 1 and 3). In table 1, the categorical variables are presented as counts and percentages, and the continuous variable is presented with mean and SD. Cox regression models were used to estimate the HRs with 95% CIs. All models were adjusted for age and sex, and tested to ensure that the assumptions for Cox regression were fulfilled. Estimated HRs were additionally interpreted in terms of the probabilistic index.^32^ SMRs with 95% CIs were calculated as the ratio of observed to expected number of deaths.^33^ The expected number of deaths was calculated using general population mortality rates from the Swedish National Board of Health and Welfare, and stratified by 5-year age groups and sex. Additionally, we performed two sensitivity regression analyses that included (1) a 1-year follow-up to test whether a shorter follow-up time impacted the Cox regression results, and (2) only psychoactive drug use (see table 2’s list of included drugs) to test whether inclusion of narcotic use other than psychoactive drug use affected the Cox regression results (online supplemental table 1). All tests were considered significant at the p <0.05 level and analyses were performed using R software, V.4.2.1 (Survival package, V.3.5.5).^34^

10.1136/jech-2023-220989.supp1 Supplementary data

**Table 3:** Standardised mortality ratios (SMRs), number of deaths and causes of death

	Homelessness	Precarious housing	Stable housing	P value
	n=637	n=1135	n=6625	All groups
Number of deaths	51 (8.0%)	38 (3.3%)	297 (4.5%)	<0.001
Mean age at death (SD)	47.5 (12.0)	42.7 (13.9)	55.6 (14.2)	<0.001
SMR (95% CI)	13.6 (10.2 to 17.9)	8.7 (6.2 to 12.0)	3.7 (3.2 to 4.1)	
Mean years of follow-up (Q1–Q3)*	3.09 (2.66–3.70)	3.15 (2.62–3.77)	3.18 (2.62–3.79)	0.050
Causes of death†				<0.001
Alcohol related	1 (2.0%)	4 (10.5%)	69 (23.2%)	
Other causes	17 (33.3%)	8 (21.1%)	112 (37.7%)	
Drug related	19 (37.3%)	16 (42.1%)	39 (13.1%)	
External causes‡	1 (2.0%)	4 (10.5%)	35 (11.8%)	
Unknown cause	13 (25.5%)	6 (15.8%)	42 (14.1%)	

*Q1–Q3: quartile 1 and quartile 3.

†See table 2 for International Classification of Diseases, 10th Revision codes of the causes of death.

‡External causes include suicide, unknown suicide, accident or assault.

## Results

### Baseline characteristics of the study population

At baseline, there were more men than women within each housing group. The proportion of women living in stable housing was 35.6% (n=2361), in homelessness it was 25.6% (n=163) and in precarious housing it was 25.6% (n=291). Among the study population, mean age for people in precarious housing at baseline was younger (M=33.9 years; SD=12.1) than those in homelessness (M=40.7 years; SD=12.6), or in stable housing (M=47.1 years; SD=14.0). All differences in sex and age were statistically significant.

Table 1 presents the descriptive statistics associated with the substance use and psychiatric variables for the people in homelessness, precarious housing and stable housing at baseline. The results in table 1 indicate that a significantly higher proportion of those experiencing homelessness had used narcotics (n=467; 73.3%) compared with those in precarious (n=681; 60.0%) or stable housing (n=1913; 28.9%). Similarly, a significantly higher proportion of those experiencing homelessness had used intravenous drugs (n=246; 38.6%) compared with those in precarious (n=165; 14.5%) or stable housing (n=574; 8.7%). Accordingly, a significantly higher proportion of those experiencing homelessness had been treated in inpatient care for SUDs (n=341; 53.5%) than those in precarious housing (n=262; 23.1%), or in stable housing (n=1071; 16.2%).

### SMRs, number of deaths and causes of death

Table 3 presents the SMRs, in addition to the number of deaths, causes of death, mean age at death and mean years of follow-up. Among the study population, the SMR for people in homelessness at baseline was 13.6 (CI 10.2 to 17.9), the SMR for people in precarious housing was 8.7 (CI 6.2 to 12.0) and the SMR for people in stable housing was 3.7 (CI 3.2 to 4.1). A higher proportion of people in homelessness at baseline died (n=51; 8.0%) than those in precarious housing (n=38; 3.3%), or in stable housing (n=297; 4.5%). Furthermore, a higher proportion of people in homelessness at baseline (n=13; 25.5%) died from unknown causes than those in precarious housing (n=6; 15.8%), or in stable housing (n=42; 14.1%). A lower proportion of people in homelessness at baseline (n=1; 2.0%) died from alcohol-related causes than those in precarious housing (n=4; 10.5%), or in stable housing (n=69; 23.2%).

### Cox regression results

**Figure 1:**
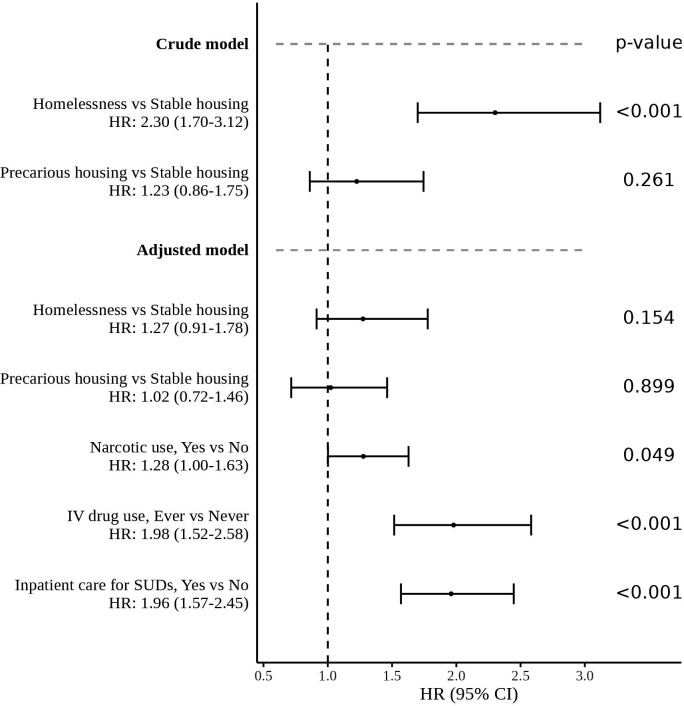
Cox regression analyses of mortality among people with substance use disorders (SUDs) in homelessness, precarious housing and stable housing (reference group).

The crude and adjusted models, with p values, are presented in figure 1.

The crude model showed that among the study population, people in homelessness at baseline had an increased risk of mortality (HR 2.30; 95% CI 1.70 to 3.12) compared with those in stable housing (figure 1). According to the probabilistic index, the probability that the survival period was longer for a person in stable housing compared with a person in homelessness was 69.7% (95% CI 63.0% to 75.7%). In the study population, people in precarious housing at baseline did not have a significant increase in risk of mortality (HR 1.23; 95% CI 0.86 to 1.75).

In the model presented in figure 1 when adjusting for narcotic use, intravenous drug use, and inpatient care for SUDs, the HR for mortality among people in homelessness was not significant (HR 1.27; 95% CI 0.91 to 1.78). Narcotic use (HR 1.28; 95% CI 1.00 to 1.63), intravenous drug use (HR 1.98; 95% CI 1.52 to 2.58) and inpatient care for SUDs (HR 1.96; 95% CI 1.57 to 2.45) were associated with significantly increased mortality among people with SUDs. In the study population, people in precarious housing at baseline did not have a significant increase in risk of mortality (HR 1.02; 95% CI 0.72 to 1.46).

### Sensitivity analyses

In a sensitivity analysis using a shorter 1-year follow-up of the main crude model, the HRs for mortality among people in homelessness (HR 3.02; 95% CI 1.94 to 4.72) and precarious housing (HR 1.72; 95% CI 1.03 to 2.85) were significant. In the adjusted model with a 1-year follow-up, the HR for mortality among people in homelessness (HR 1.70; 95% CI 1.04 to 2.80) became significant. The HR for precarious housing (HR 1.44; 95% CI 0.86 to 2.40) was still not significant.

In another sensitivity analysis adjusting for selective psychoactive drug use, the HRs for mortality among people in homelessness remained similar to the main results (online supplemental table 1).

## Discussion

### Summary of key findings

In the present study, homelessness at baseline was associated with an increased risk of mortality which supports previous studies.^2 8–15^ The relative mortality risk for people in homelessness decreased after adjusting for narcotic use, intravenous drug use and inpatient care for SUDs. This aligns with earlier research showing that alcohol and drug use contribute to excess mortality in this population.^15^ A higher proportion of people in homelessness had psychiatric comorbidities, in addition to narcotic use, intravenous drug use and inpatient care for SUDs, than people in precarious housing or stable housing. However, a lower proportion of people in homelessness used alcohol compared with people in precarious housing or stable housing.

SMRs showed that mortality among people in homelessness with SUDs was 13.6 times higher than the general population, and 3.7 times higher in people in stable housing with SUDs. The estimated SMR for people in homelessness with SUDs was higher than previous estimates among people in homelessness, which range between 2.0 and 6.7^1 2 9–17^; however, there are considerable differences between the study populations.

Similar to earlier studies, narcotic use,^15 18^ intravenous drug use^3 22–24^ and inpatient care, sometimes referred to as a treatment for SUDs,^15^ were associated with excess mortality.

### Implications for policy and practice

In terms of policy and practice implications, the results indicate a need for addiction care interventions to support people in homelessness with SUDs. The results support international research^35^ and guidance^36^ on health and social responses to homelessness and substance use, calling for integrated strategies connecting services (eg, addiction care, housing support, psychosocial services, among others) that are targeted to the individual’s needs.

Using the shorter follow-up time of 1 year in the sensitivity analyses showed an increased mortality risk among the people in homelessness. A shorter follow-up provides potentially more up-to-date information on homelessness which this study demonstrated is a risk factor for mortality.

### Strengths and limitations

A strength of this study is its use of the quality register, which provides unique individual-level data on homelessness from addiction care. It is challenging to access cohort data on homelessness since it is not included in national health data registers. The quality register provided access to a larger, population-based cohort which addresses some of the limitations of earlier research based on smaller samples of people participating in specific healthcare programmes for homelessness,^25^ or samples of community-based shelter records which do not account for different types of homelessness (ie, rough sleeping, temporary lodgings or institutions).^2 8 9 15^

The present study’s analysis focused on the psychiatric diagnoses in relation to addiction care and housing variables at baseline and did not account for changes over the follow-up period that may have impacted mortality. It is likely that many of those in the study population, particularly those experiencing homelessness, had somatic health comorbidities that may have contributed to increased mortality.^1 3 6 7 20^ Consequently, the results likely include physical health conditions that were beyond the scope of this study.

Regarding the self-reported variables, earlier research has shown that self-reported data on substance use collected at treatment intake is often accurate.^37–39^ Severe narcotic users are more likely to under-report the frequency of substance use rather than deny it.^40^ A meta-analysis indicated that false omission rates are generally low, especially if there are no consequences and if people are asked about longer reference periods.^41^ Moreover, self-classifications of housing status may not be consistent if patients have different understandings of what constitutes homelessness.

### Future directions

There were insufficient data to stratify our analyses according to sex but in future studies it is advisable to investigate differences between the sexes in terms of their experiences of housing status when living with SUDs, and how these differences may impact mortality. Furthermore, the large age difference between groups at baseline limited the interpretation of differences in mean age at death in a meaningful way.

The duration of homelessness could not be analysed because this information is not available in the quality register. There are limited national and regional statistics on the length of time a person spends in homelessness or other types of housing status. A report from the Swedish National Board of Health and Welfare estimated that more than two-thirds of people in homelessness in Sweden have been in homelessness for a year or longer, and approximately 3000 have been in homelessness for at least 10 years.^5^ However, significant variation in homelessness duration has been reported in different countries and regions.^4 5^

## Conclusion

Homelessness at baseline was associated with excess risk of mortality in the study population with SUDs. The excess risk of mortality decreased after adjusting for narcotic use, intravenous drug use and inpatient care for SUDs. The findings underscore the need for targeted interventions to support individuals experiencing both homelessness and SUDs, with the objective of reducing the elevated risk of mortality within this vulnerable population.

## Data Availability

Data are available upon reasonable request. Register data are available upon reasonable request from the Swedish National Addiction Care Quality Register.

## References

[1] Fazel S , Geddes JR , Kushel M . The health of homeless people in high-income countries: descriptive epidemiology, health consequences, and clinical and policy recommendations. Lancet 2014;384:1529–40. 10.1016/S0140-6736(14)61132-6 25390578 PMC4520328

[2] Nielsen SF , Hjorthøj CR , Erlangsen A , et al . Psychiatric disorders and mortality among people in homeless shelters in Denmark: a nationwide register-based cohort study. Lancet 2011;377:2205–14. 10.1016/S0140-6736(11)60747-2 21676456

[3] Aldridge RW , Story A , Hwang SW , et al . Morbidity and mortality in homeless individuals, prisoners, sex workers, and individuals with substance use disorders in high-income countries: a systematic review and meta-analysis. Lancet 2018;391:241–50. 10.1016/S0140-6736(17)31869-X 29137869 PMC5803132

[4] Fondation Abbé Pierre and FEANTSA . Seventh overview of housing exclusion in Europe. Brussels: FEANTSA, 2022.

[5] The Swedish National Board of Health and Welfare [Socialstyrelsen] . Homelessness 2017 – scope and characteristics [Hemlöshet 2017 - Omfattning och Karaktär]. 2017.

[6] Vickery KD , Winkelman TNA , Ford BR , et al . Trends in trimorbidity among adults experiencing homelessness in Minnesota, 2000-2018. Med Care 2021;59:S220–7. 10.1097/MLR.0000000000001435 33710099 PMC7958979

[7] Seastres RJ , Hutton J , Zordan R , et al . Long-term effects of homelessness on mortality: a 15-year Australian cohort study. Aust N Z J Public Health 2020;44:476–81. 10.1111/1753-6405.13038 32955766

[8] Feodor Nilsson S , Laursen TM , Hjorthøj C , et al . Homelessness as a predictor of mortality: an 11-year register-based cohort study. Soc Psychiatry Psychiatr Epidemiol 2018;53:63–75. 10.1007/s00127-017-1456-z 29124292

[9] Stenius-Ayoade A , Haaramo P , Kautiainen H , et al . Mortality and causes of death among homeless in Finland: a 10-year follow-up study. J Epidemiol Community Health 2017;71:841–8. 10.1136/jech-2017-209166 28739837

[10] Nordentoft M , Wandall-Holm N . 10 year follow up study of mortality among users of hostels for homeless people in Copenhagen. BMJ 2003;327:81. 10.1136/bmj.327.7406.81 12855527 PMC164916

[11] Hibbs JR , Benner L , Klugman L , et al . Mortality in a cohort of homeless adults in Philadelphia. N Engl J Med 1994;331:304–9. 10.1056/NEJM199408043310506 8022442

[12] Hwang SW , Wilkins R , Tjepkema M , et al . Mortality among residents of shelters, rooming houses, and hotels in Canada: 11 year follow-up study. BMJ 2009;339:b4036. 10.1136/bmj.b4036 19858533 PMC2767481

[13] Barrow SM , Herman DB , Córdova P , et al . Mortality among homeless shelter residents in New York City. Am J Public Health 1999;89:529–34. 10.2105/ajph.89.4.529 10191796 PMC1508869

[14] Morrison DS . Homelessness as an independent risk factor for mortality: results from a retrospective cohort study. Int J Epidemiol 2009;38:877–83. 10.1093/ije/dyp160 19304988

[15] Beijer U , Andreasson S , Agren G , et al . Mortality and causes of death among homeless women and men in Stockholm. Scand J Public Health 2011;39:121–7. 10.1177/1403494810393554 21247970

[16] Beijer U , Andréasson A , Agren G , et al . Mortality, mental disorders and addiction: a 5-year follow-up of 82 homeless men in Stockholm. Nord J Psychiatry 2007;61:363–8. 10.1080/08039480701644637 17990198

[17] Nusselder WJ , Slockers MT , Krol L , et al . Mortality and life expectancy in homeless men and women in Rotterdam: 2001-2010. PLoS One 2013;8:e73979. 10.1371/journal.pone.0073979 24098329 PMC3788767

[18] Baggett TP , Chang Y , Singer DE , et al . Tobacco-, alcohol-, and drug-attributable deaths and their contribution to mortality disparities in a cohort of homeless adults in Boston. Am J Public Health 2015;105:1189–97. 10.2105/AJPH.2014.302248 25521869 PMC4431083

[19] Beijer U , Andréasson S . Gender, hospitalization and mental disorders among homeless people compared with the general population in Stockholm. Eur J Public Health 2010;20:511–6. 10.1093/eurpub/ckq033 20371499

[20] Tweed EJ , Thomson RM , Lewer D , et al . Health of people experiencing co-occurring homelessness, imprisonment, substance use, sex work and/or severe mental illness in high-income countries: a systematic review and meta-analysis. J Epidemiol Community Health 2021;75:1010–8. 10.1136/jech-2020-215975 33893182 PMC8458085

[21] Nilsson SF , Laursen TM , Hjorthøj C , et al . Risk of homelessness after discharge from psychiatric wards in Denmark: a nationwide register‐based cohort study. Acta Psychiatr Scand 2019;140:477–89. 10.1111/acps.13082 31385289

[22] Linton SL , Celentano DD , Kirk GD , et al . The longitudinal association between homelessness, injection drug use, and injection-related risk behavior among persons with a history of injection drug use in. Drug Alcohol Depend 2013;132:457–65. 10.1016/j.drugalcdep.2013.03.009 23578590 PMC3926693

[23] Arum C , Fraser H , Artenie AA , et al . Homelessness, unstable housing, and risk of HIV and hepatitis C virus acquisition among people who inject drugs: a systematic review and meta-analysis. Lancet Public Health 2021;6:e309–23. 10.1016/S2468-2667(21)00013-X 33780656 PMC8097637

[24] Mathers BM , Degenhardt L , Bucello C , et al . Mortality among people who inject drugs: a systematic review and meta-analysis. Bull World Health Organ 2013;91:102–23. 10.2471/BLT.12.108282 23554523 PMC3605003

[25] Funk AM , Greene RN , Dill K , et al . The impact of homelessness on mortality of individuals living in the United States: a systematic review of the literature. J Health Care Poor Underserved 2022;33:457–77. 10.1353/hpu.2022.0035 35153234

[26] Baggett TP , Hwang SW , O’Connell JJ , et al . Mortality among homeless adults in Boston: shifts in causes of death over a 15-year period. JAMA Intern Med 2013;173:189–95. 10.1001/jamainternmed.2013.1604 23318302 PMC3713619

[27] Roncarati JS , Baggett TP , O’Connell JJ , et al . Mortality among unsheltered homeless adults in Boston, Massachusetts, 2000-2009. JAMA Intern Med 2018;178:1242–8. 10.1001/jamainternmed.2018.2924 30073282 PMC6142967

[28] Hwang SW . Is homelessness hazardous to your health? Obstacles to the demonstration of a causal relationship. Can J Public Health 2002;93:407–10. 10.1007/BF03405026 12448860 PMC6980210

[29] Montgomery AE , Szymkowiak D , Culhane D . Gender differences in factors associated with unsheltered status and increased risk of premature mortality among individuals experiencing homelessness. Womens Health Issues 2017;27:256–63. 10.1016/j.whi.2017.03.014 28456453

[30] Better Addiction Care . Årsrapport 2021 [annual report 2021]. Stockholm Stockholm County Health District (SLSO); 2021.

[31] World Health Organization (WHO) . International statistical classification of diseases and related health problems. 10th edn. 2019.

[32] De Neve J , Gerds TA . On the interpretation of the hazard ratio in Cox regression. Biom J 2020;62:742–50. 10.1002/bimj.201800255 30623465

[33] Ulm K . A simple method to calculate the confidence interval of a standardized mortality ratio (SMR). Am J Epidemiol 1990;131:373–5. 10.1093/oxfordjournals.aje.a115507 2296988

[34] R Core Team . R: A language and environment for statistical computing. Vienna, Austria: R Foundation for Statistical Computing, 2022.

[35] Miler JA , Carver H , Masterton W , et al . What treatment and services are effective for people who are homeless and use drugs? A systematic 'review of reviews. PLoS One 2021;16:e0254729. 10.1371/journal.pone.0254729 34260656 PMC8279330

[36] European Monitoring Centre for Drugs and Drug Addiction [EMCDDA] . Health and social responses to drug problems: a European guide. 2023.

[37] Denis C , Fatséas M , Beltran V , et al . Validity of the self-reported drug use section of the addiction severity index and associated factors used under naturalistic conditions. Subst Use Misuse 2012;47:356–63. 10.3109/10826084.2011.640732 22216906

[38] Wish ED , Hoffman JA , Nemes S . The validity of self-reports of drug use at treatment admission and at follow-up: comparisons with urinalysis and hair assays. NIDA Res Monogr 1997;167:200–26. 9243563

[39] Johnson TP . Sources of error in substance use prevalence surveys. Int Sch Res Notices 2014;2014:923290. 10.1155/2014/923290 27437511 PMC4897110

[40] Morral AR , McCaffrey D , Iguchi MY . Hardcore drug users claim to be occasional users: drug use frequency underreporting. Drug Alcohol Depend 2000;57:193–202. 10.1016/s0376-8716(99)00048-4 10661670

[41] Bharat C , Webb P , Wilkinson Z , et al . Agreement between self‐reported illicit drug use and biological samples: a systematic review and Meta‐Analysis. Addiction 2023;118:1624–48. 10.1111/add.16200 37005867

