# Supplementary material for: Excess mortality among people in homelessness with substance use disorders: a Swedish cohort study
Source: J Epidemiol Community Health. 2024 May 21;78(8):473–8. doi: 10.1136/jech-2023-220989 (PMC11287526; doi:10.1136/jech-2023-220989)
Supplement: Supplementary data [file jech-2023-220989supp001.pdf]

Supplementary Table 1 Cox regression analyses of mortality among people with SUDs in homelessness, precarious housing, and stable housing (adjusting for selective psychoactive drug use)

	Hazard ratio (95% CI)	
		One-year follow-up
Homelessness vs Stable housing	1.24 (0.89 – 1.73)	1.62 (0.98 – 2.67)
Precarious housing vs Stable housing	1.03 (0.72 – 1.47)	1.44 (0.86 – 2.39)
Selective psychoactive drug use, Yes vs No	1.62 (1.23 – 2.14)**	1.74 (1.14 – 2.66)*
IV drug use, Ever vs Never	1.83 (1.39 – 2.40)**	1.96 (1.30 – 2.98)**
Inpatient care for SUDs, Yes vs No	1.92 (1.54 – 2.40)**	1.66 (1.16 – 2.38)**

Note. All models were adjusted for age and sex; SUDs: substance use disorders; * $p<0.05$; ** $p<0.01$.
